# Combined low-pass whole genome and targeted sequencing in liquid biopsies for pediatric solid tumors

**DOI:** 10.1038/s41698-023-00357-0

**Published:** 2023-02-20

**Authors:** Eirini Christodoulou, Venkata Yellapantula, Katrina O’Halloran, Liya Xu, Jesse L. Berry, Jennifer A. Cotter, Anya Zdanowicz, Leo Mascarenhas, James F. Amatruda, Dejerianne Ostrow, Moiz Bootwalla, Xiaowu Gai, Fariba Navid, Jaclyn A. Biegel

**Affiliations:** 1 Department of Pathology and Laboratory Medicine, Children’s Hospital Los Angeles, Los Angeles, CA USA; 2 Keck School of Medicine at the University of Southern California, Los Angeles, CA USA; 3 Cancer and Blood Disease Institute, Children’s Hospital Los Angeles, Los Angeles, CA USA; 4 The Vision Center, Children’s Hospital Los Angeles, Los Angeles, CA USA

**Keywords:** Molecular medicine, Cancer genomics

## Abstract

We designed a liquid biopsy (LB) platform employing low-pass whole genome sequencing (LP-WGS) and targeted sequencing of cell-free (cf) DNA from plasma to detect genome-wide copy number alterations (CNAs) and gene fusions in pediatric solid tumors. A total of 143 plasma samples were analyzed from 19 controls and 73 patients, including 44 bone or soft-tissue sarcomas and 12 renal, 10 germ cell, five hepatic, and two thyroid tumors. cfDNA was isolated from plasma collected at diagnosis, during and after therapy, and/or at relapse. Twenty-six of 37 (70%) patients enrolled at diagnosis without prior therapy (radiation, surgery, or chemotherapy) had circulating tumor DNA (ctDNA), based on the detection of CNAs from LP-WGS, including 18 of 27 (67%) patients with localized disease and eight of 10 (80%) patients with metastatic disease. None of the controls had detectable somatic CNAs. There was a high concordance of CNAs identified by LP-WGS to CNAs detected by chromosomal microarray analysis in the matching tumors. Mutations identified in tumor samples with our next-generation sequencing (NGS) panel, OncoKids®, were also detected by LP-WGS of ctDNA in 14 of 26 plasma samples. Finally, we developed a hybridization-based capture panel to target *EWSR1* and *FOXO1* fusions from patients with Ewing sarcoma or alveolar rhabdomyosarcoma (ARMS), respectively. Fusions were detected in the plasma from 10 of 12 patients with Ewing sarcoma and in two of two patients with ARMS. Combined, these data demonstrate the clinical applicability of our LB platform to evaluate pediatric patients with a variety of solid tumors.

## Introduction

Pediatric solid tumors encompass a heterogeneous group of rare malignancies that constitute approximately 40% of all childhood cancers^1^. Among them, soft-tissue tumors including embryonal tumors, germ cell tumors, and renal tumors account for approximately 10% of cases, whereas bone and soft-tissue sarcomas comprise ~20% of all cases^2–4^. The cytogenetic and molecular genetic characterization of pediatric solid tumors is used clinically to aid in diagnosis and determine prognosis, and in certain cases, guide treatment^5^. Identification of germline copy number alterations (CNAs) and sequence variants in cancer predisposition genes are also important for patient management, specifically to address the risk for second malignancies in the patient or cancer in a family member.

Liquid biopsy (LB) assays for pediatric patients with solid tumors have the potential to transform patient care by providing a less invasive alternative to diagnostic biopsies for identifying genomic aberrations that can inform diagnosis, risk stratification, and therapeutic options as well as enable earlier detection of disease progression compared with conventional radiographic imaging^6–11^. The clinical effectiveness of plasma-based LB approaches, however, depends on optimizing the detection of circulating tumor DNA (ctDNA) fragments and distinguishing them from cfDNA derived predominantly from hematopoietic cells^12,13^.

Recent advances in LB test development have focused primarily on adult-type malignant epithelial tumors defined by a spectrum of recurrent dominant activating mutations^14–16^. Pediatric tumors, however, are more often characterized by CNAs, loss of tumor suppressor genes, epigenetic modifications, and large-scale structural rearrangements with breakpoints that are variable and located in the intronic regions of the genome^5,17,18^. Furthermore, the incidence of pediatric solid tumors is low, and the number of histologic, genomic, and clinical subtypes is large, suggesting that the combined use of a pan-cancer assay and a target-specific approach may be better suited for clinical applications. Most importantly, the clinical development of LB assays for pediatric solid tumor patients has been limited by the requirement for relatively large volumes of blood, urine, or cerebrospinal fluid (CSF) to isolate sufficient amounts of cancer-derived nucleic acids for analysis.

The feasibility of employing NGS and droplet digital PCR-based assays utilizing cfDNA derived from CSF, plasma, or the aqueous humor of the eye for pediatric central nervous system (CNS) tumors, solid tumors, or retinoblastoma, respectively, has recently been described^10,19–27^. In the diagnostic setting, ultra-low-pass WGS (ULP-WGS) analysis of plasma ctDNA was effectively used to distinguish malignant peripheral nerve sheath tumors (MPNST) from the benign lesion, plexiform neurofibroma (PN), in patients with the neurofibromatosis type 1 (NF1) cancer predisposition syndrome^28^. By using in silico enrichment of short cfDNA fragments and copy number analysis, MPNST samples were found to be enriched with ctDNA when compared to PN, and treatment response was correlated with the ctDNA-derived estimate of tumor burden^28^. To assess response to treatment, Liu et al. assessed measurable residual disease (MRD) in 123 children with medulloblastoma using LP-WGS of cfDNA derived from CSF^10^. The presence of MRD in the CSF was found to be associated with a higher risk of relapse. The presence of ctDNA in plasma has been proposed as a prognostic biomarker in pediatric solid tumors (reviewed in ref. ^7^), and targeted detection of *EWSR1* and *FOXO1* fusions in Ewing sarcoma and alveolar rhabdomyosarcoma (ARMS) has been demonstrated in small cohorts of patients^29,30^.

The Precision Medicine Program in Pediatric and Adolescent Patients with Recurrent Malignancies (MAPPYACTS) employed whole exome sequencing (WES) of tumor tissue and cfDNA from plasma to identify targeted therapies in patients with relapsed/recurrent non-CNS solid tumors^31^. Notably, 57% of the somatic SNVs were detected in both the tumor and the cfDNA, whereas 31% were specific to the tumor and 11% were specific to the cfDNA, reflecting tumor heterogeneity, or possibly technical limitations of the assays^31^. Significantly more mutations were detected in patients with metastatic (66%) vs localized (47%) disease.

The studies published to date have demonstrated the feasibility of using cfDNA from plasma to detect CNAs, sequence variants, and/or gene fusions, but have focused on disease-specific cohorts, or patients with recurrent/refractory disease. The aim of the present study was to evaluate the potential clinical applicability of a plasma-based LB assay for newly diagnosed and relapsed pediatric patients with a variety of solid tumors. In this approach, LP-WGS was performed using cfDNA to detect CNAs and mutations. Targeted panel-based sequencing of cfDNA was also performed to identify specific gene fusions. Clinical validation of these assays will allow for their implementation in a prospective pan-cancer setting as an aid in diagnosis and to monitor response to therapy.

## Results

### Patient clinical characteristics

Patients were deemed eligible for this study if they had a newly diagnosed or recurrent malignant bone or soft-tissue sarcoma or germ cell, hepatic, thyroid, or renal tumor. Written informed consent (and assent when appropriate) was obtained from all patients, their parents, or a legal guardian to participate in this study under a Children’s Hospital Los Angeles Institutional Review Board-approved protocol (CHLA-19-00146). Written informed consent was also obtained for non-oncologic controls, their parents, or a legal guardian to participate in this study under a Children’s Hospital Los Angeles Institutional Review Board-approved protocol (CHLA-19-00230).

One hundred and forty-three samples from 73 eligible patients and 19 non-oncologic controls were analyzed. The patients were diagnosed as having sarcoma (*n* = 44), renal tumor (*n* = 12), hepatic tumor (*n* = 5), malignant germ cell tumor (*n* = 10), or thyroid carcinoma (*n* = 2). The age of the patients ranged from six months to 28 years (median 12 years) and there were 37 males and 36 females (Table 1). The median age of the controls was 11 years (Supplemental Table 1). A median of two samples were analyzed per patient (range, 1–6). Forty-eight of the patients were enrolled at diagnosis, although 11 had received chemotherapy and/or undergone surgery prior to obtaining a blood sample for the study. Thirty-five of the patients presented with localized disease. Twenty-five patients were enrolled at the time of a recurrence (two with local and 23 with distant disease). Six patients had received chemotherapy and/or had undergone surgery for relapse prior to enrollment. None of the patients received prior radiation therapy. Two patients had surgery and chemotherapy prior to study enrollment (Table 1 and Supplemental Table 1). Samples were collected at the time of initial diagnosis or recurrence, during therapy, at the time of recurrence/progression, where applicable, and during follow-up (i.e., after treatment).

**Table 1:** Patient characteristics.

	Total (*n* = 73)	Enrolled at initial diagnosis (*n* = 48)		Enrolled at recurrence (*n* = 25)	
		Stage		Type of recurrence	
		Localized (*n* = 35)	Metastatic (*n* = 13)	Local (*n* = 2)	Distant (*n* = 23)
Age (yrs)					
0.5-<6	13	9	2	0	2
6-<12	21	11	3	1	6
12-<18	25	13	5	6	1
>18	14	2	3	0	9
Gender					
Male	37	16	7	2	12
Female	36	19	6	0	11
Tumor Type					
Sarcoma	44	20	9	1	14
Ewing sarcoma	15	7	6	0	2
Osteosarcoma	20	7	2	0	11
Alveolar RMS	3	2	1	0	0
Embryonal RMS	2	1	0	1	0
Other sarcoma	4	3	0	0	1
Renal Tumor	12	6	1	0	5
Wilms tumor	9	5	1	0	3
Other	3	1	0	0	2
Hepatic Tumors	5	5	0	0	0
Hepatoblastoma	3	3	0	0	0
Embryonal sarcoma liver	2	2	0	0	0
Malignant germ cell tumors	10	4	2	1	3
Thyroid carcinoma	2	0	1	0	1
Systemic therapy prior to enrollment					
Yes	8	3	1	1	3
No	65	32	12	1	20
Definitive surgery prior to enrollment					
Yes	11	7	2	1	1
No	62	28	11	1	22

### Detection of copy number alterations in plasma from pediatric solid tumor patients

**Fig. 1:**
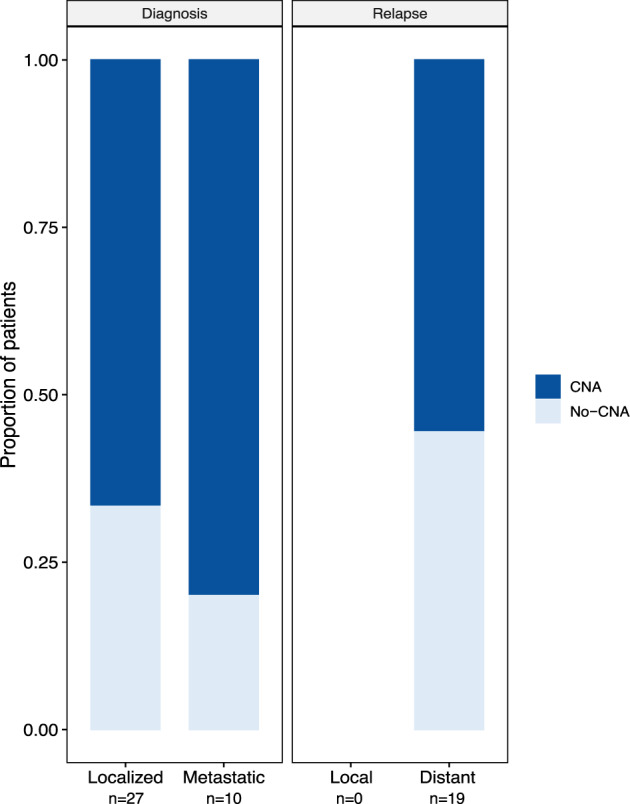
Copy number alteration detection in liquid biopsies of pediatric solid tumor patients. Summary of copy number alteration (CNA) detection and disease status at initial diagnosis and relapse in treatment naïve patients (i.e., no definitive surgery, radiation, or chemotherapy). CNAs were detected in the plasma from 18 of 27 (67%) and eight of 10 (80%) newly diagnosed patients with localized disease and metastatic disease, respectively. CNAs were detected in the plasma of 10 of 19 (53%) patients with distant recurrence of the disease. Dark blue- CNA positive, Light blue- CNA negative.

The presence of ctDNA was determined by LP-WGS, evidenced by CNAs detected in plasma. To improve the sensitivity of detecting CNAs and to take advantage of the higher depth of coverage achieved, we used an in silico size selection approach to enrich for fragments between 90–150 bp in length (Supplemental Fig. 1)^32^. Positivity rates were determined by analyzing true diagnostic samples (defined as samples obtained prior to chemotherapy or definitive surgery) either at initial diagnosis or relapse at the time of enrollment (Table 2). There was detectable ctDNA by LP-WGS in 26 of 37 (70%) diagnostic samples and 10 of 19 (43%) relapse samples (Fig. 1 and Table 2). Eighteen of 27 (67%) patients were diagnosed with localized disease and eight of 10 (80%) patients with metastatic disease had CNAs detected by LP-WGS. CNAs were detectable in 10 of 19 (53%) patients enrolled at relapse with distant disease (Fig. 1 and Table 2). Neither of the two patients with local recurrences had CNAs detected in the LB. For the non-oncologic controls, 16 samples were negative and three demonstrated previously identified germline deletions, and were therefore subsequently excluded from further analysis (Supplemental Table 1).

**Table 2:** Detection of copy number alterations (CNA) in the plasma by histologic subtype.

	CNA positive (%)	Enrolled at initial diagnosis (*n* = 37)		Enrolled at recurrence (*n* = 19)
		Stage		
		Localized (*n* = 27)	Metastatic (*n* = 10)	
CNA positive (%)	36 (64%)	18 (67%)	8 (80%)	10 (53%)
Tumor Type				
Sarcoma (*n* = 37)	21 (57%)	11 (61%)	6 (75%)	4 (36%)
Ewing Sarcoma (*n* = 13)	9 (69%)	4 (57%)	4 (80%)	1 (100%)
Osteosarcoma (*n* = 17)	9 (53%)	4 (67%)	2 (100%)	3 (33%)
Alveolar RMS (*n* = 2)	1 (50%)	1 (50%)	0	0
Embryonal RMS (*n* = 1)	0	0	0	0
Other sarcoma (*n* = 4)	2 (50%)	2 (50%)	0	0
Renal Tumor (*n* = 9)	8 (89%)	2 (67%)	1 (100%)	5 (100%)
Wilms tumor (*n* = 7)	6 (86%)	2 (67%)	1 (100%)	3 (100%)
Other (*n* = 2)	2 (100%)	0	0	2 (100%)
Hepatic Tumor (*n* = 4)	3 (75%)	3 (75%)	0	0
Hepatoblastoma (*n* = 3)	2 (67%)	2 (67%)	0	0
Embryonal sarcoma liver (*n* = 1)	1 (100%)	1 (100%)	0	0
Malignant Germ Cell Tumor (*n* = 5)	4 (80%)	2 (100%)	1 (100%)	1 (50%)
Thyroid Carcinoma (*n* = 1)	0	0	0	0

Next, we tested whether characteristic copy number changes for each tumor type could be detected using cfDNA (Table 2). Marked genomic instability, characteristic of osteosarcoma, was readily detectable by LP-WGS in nine of 17 (53%) patients analyzed either at diagnosis or relapse (Supplemental Fig. 2a). Nine of 13 (69%) Ewing sarcoma patients (Supplemental Fig. 2b) had detectable CNAs, primarily gain of chromosome 8 (seven of 13 samples), a frequent abnormality observed in this disease^5^. CNAs were also detectable in three of four (75%) newly diagnosed renal tumor patients, and five of five (100%) patients at relapse with a renal tumor **(**Supplemental Fig. 2c). Moreover, all three patients with newly diagnosed germ cell tumor and one of two (50%) patients with germ cell tumor at relapse (Supplemental Fig. 2d) had detectable CNAs (Table 2). The most common genetic aberrations in germ cell tumors were chromosome 1 and 12p gains, characteristic of this diesase^5^. Three of four (75%) of the patients with hepatic tumors, including two with hepatoblastoma and one with an embryonal sarcoma of the liver, had detectable CNAs at diagnosis (Supplemental Fig. 2e). One patient with a peripheral malignant nerve sheath tumor (MPNST), a patient with ARMS and one patient with synovial cell sarcoma had CNAs detected by LP-WGS at diagnosis (Supplemental Fig. 2f). Neither of the two patients with thyroid tumors had detectable CNAs (Supplemental Table 1). CNAs were observed in two of 11 patients who had chemotherapy and/or surgery prior to enrollment at diagnosis, one with a Wilms tumor (patient 55) and one with a germ cell tumor (patient 71), Interestingly, patient 55 was newly diagnosed with a localized Wilms tumor and had undergone nephrectomy prior to enrollment. Two of six patients who received chemotherapy prior to enrollment at relapse, one with osteosarcoma (patient 10) and the other with Ewing sarcoma (patient 35), had detectable CNAs in the LB (Supplemental Table 1).

The CNA profiles of the primary, relapse, or metastatic tumors generated from clinical CMA assays (CytoScanHD or OncoScan) were available for 59 patients, including patients who received therapy prior to enrollment (Supplemental Table 1). Forty-nine of the 59 (83%) had abnormal CNA profiles in the tumor tissue. Thirty-four of the 49 (69%) informative cases had abnormal CNA profiles in the plasma by LP-WGS. The CNAs identified by LP-WGS for 21 patients with high-tumor fraction in ctDNA (>10%) were highly correlated with the CMA profiles of the matched tumor samples (Pearson’s correlation coefficient, *r*^2^ > 0.7) (Supplemental Fig. 3a, b). For example, LP-WGS from the plasma and CMA analysis from the primary tumor in a patient with metastatic Wilms tumor (patient 58) revealed nearly identical CN changes (*r*^2^ value of 0.96) affecting different chromosomes (1q gain, 7p loss, 7q gain, 10 gain, 11q loss, 12 and 20 gain) (Supplemental Fig. 3c, d). There was also a group of six patients with matching LP-WGS and CMA data but an *r*^2^ value lower than 0.7. For example, case 2 was osteosarcoma with complex chromosomal rearrangements affecting almost all chromosomes. The CNA profiles from CMA and LP-WGS of ctDNA had an *r*^2^ of 0.5 (Supplemental Fig. 3e, f). The majority of CNAs were the same in both profiles suggesting that they originated from the same clone(s) thus the low *r*^2^ value did not reflect the true biologic concordance.

**Fig. 2:**
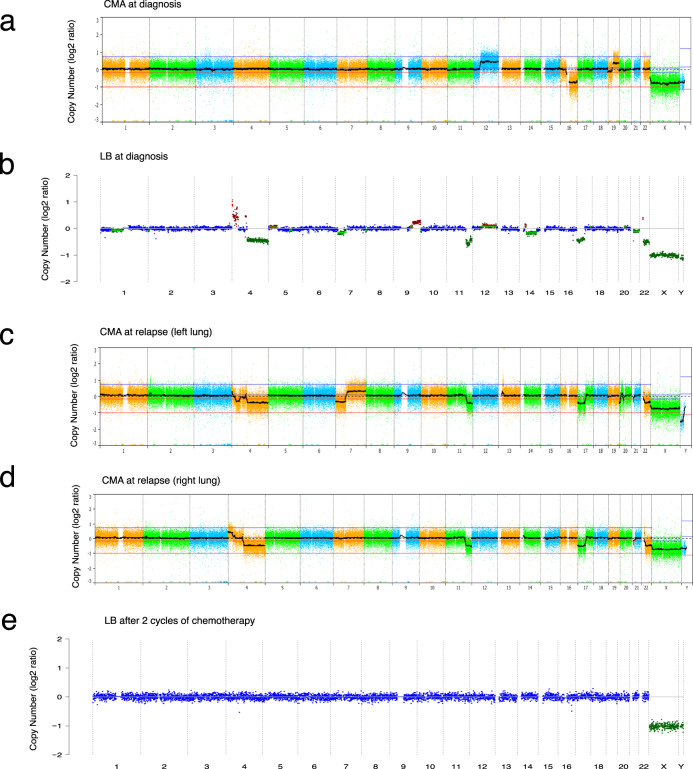
Plasma CNA profile of a patient with Wilms tumor mimics resected residual lung nodules following neoadjuvant chemotherapy. **a** Chromosomal microarray analysis (CMA) plot of the primary renal tumor resected at the time of diagnosis from a male patient with Stage IV anaplastic Wilms tumor (patient 56) demonstrates LOH for a region of the short arm of chromosome 11, a gain of 12q, loss of 16q; and gain of 19q. **b** CNA detection at baseline in the liquid biopsy sample reveals distinct copy number (CN) changes when compared to the primary tumor. Specifically, chromosome 1p loss, 4p gain, 4q loss, 5p gain, 7p loss, 9q gain, 11q loss, 12q gain, 14q loss, 17p loss, and chromosomes 21 and 22 loss. The calculated tumor fraction (TFx) was 53%. **c** CMA profile of CN changes from the residual lung nodules (left), and **d** (right) resected after two cycles of chemotherapy with chromosome 4p gain, 4q loss, 7q gain (left lung mass), 11q loss, 17p loss, chromosome 22 loss. **e** CNA profile in the plasma after two cycles of chemotherapy showing a flat profile. The calculated TFx was 0%. For each serial LB sample, log2ratio is plotted on the y-axis against chromosome numbers 1-22, X, Y. Red indicates CN gain, blue indicates CN neutral, and green indicates CN loss.

In a patient with metastatic anaplastic Wilms tumor (56), the CNA profile from plasma-derived cfDNA was distinct from that of the primary tumor (*r*^2^ < 0.7) (Fig. 2). The primary tumor demonstrated loss of heterozygosity (LOH) of the distal region of the short arm of chromosome 11, as well as gain of 12q, loss of 16q, and gain of 19q. The cfDNA profile at diagnosis showed a partial gain of chromosome 4p, loss of 4q, loss of 7p, gain of 9q, and loss of 11q, 17p, and 22, which was distinctly different from the primary tumor but consistent with the CNA profiles that were later seen in the two non-responding residual lung nodules resected following two cycles of neoadjuvant chemotherapy (Fig. 2a–c and Supplemental Table 1). Homozygosity of 11p was supported by the LP-WGS data as well as by CMA analysis of the lung metastases, confirming the common genetic origin for all three tumors. Mutation analysis of the LP-WGS data from cfDNA demonstrated the identical *TP53* mutation that was detected by OncoKids® NGS analysis of the two metastases in the lungs, which was not present in the primary tumor. The plasma samples obtained after the two cycles of chemotherapy prior to resection of the residual lung nodules showed no detectable CNAs, including the *TP53* mutation (Fig. 2d). Taken together, these findings suggest that the cfDNA profile more accurately reflected the molecular profile of the metastatic clone than the primary tumor.

### LP-WGS reveals the presence of ctDNA prior to clinical recurrence

**Fig. 3:**
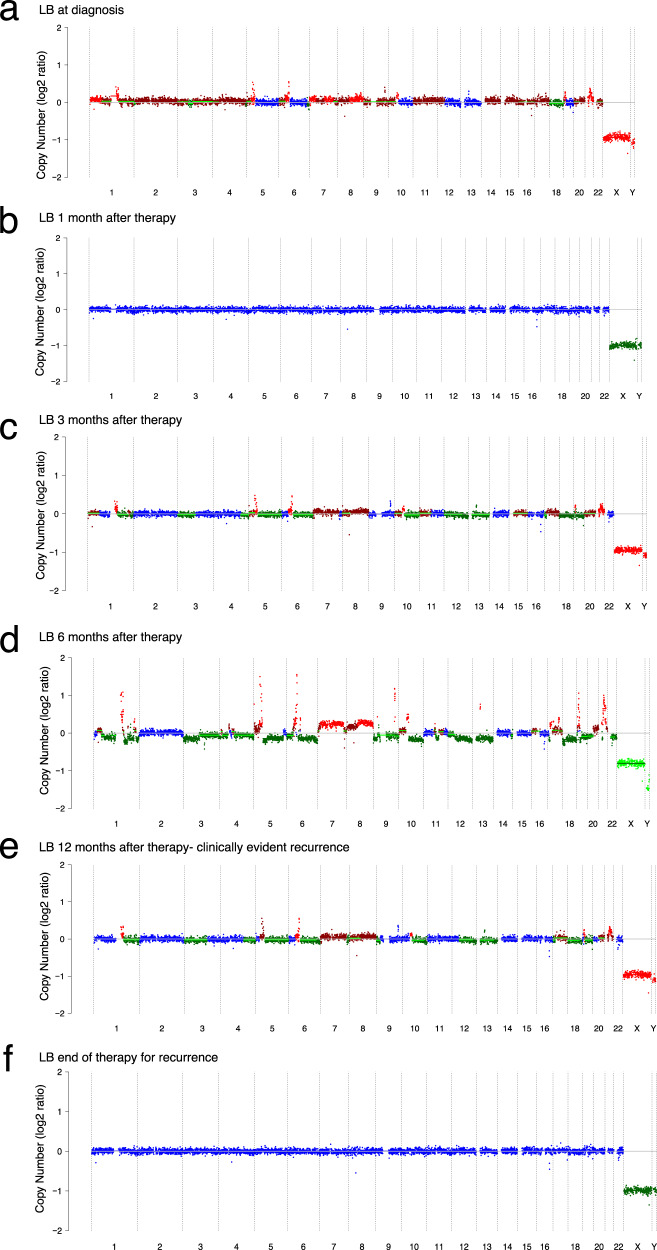
Detection of recurrence in the liquid biopsy of a patient with osteosarcoma. **a** CNA profile in the plasma at the time of diagnosis in a patient with metastatic osteosarcoma (patient 1) showing copy number changes in chromosomes 1q, 5p, 6p, 19p, and 21. The calculated tumor fraction (TFx) was 6%**. b** CNA profile with no detectable alterations one month after completion of planned therapy, including surgical resection of the primary tumor, bilateral metastasectomy of residual lung lesions, and chemotherapy. The calculated TFx was 0%. **c** CNA detection at three months and **d** 6 months after completion of therapy matches copy number changes observed at diagnosis without any clinical/imaging evidence of disease recurrence. The calculated TFx was 6.5% and 20%, respectively. **e** CNA detection at 12 months after completion of therapy at the time patient presented with symptoms of recurrent disease confirmed by imaging at a distant bony site. The calculated TFx was 9%**. f** CNA profile with no detectable alterations at the end of therapy. The calculated TFx was 0%. For each serial sample, the estimated ichorCN log2 ratio is plotted on the y-axis, and chromosomes 1-22, X, and Y are shown on the x-axis. Red indicates CN gain, blue indicates CN neutral, and green indicates CN loss.

We then examined whether recurrence was detectable in the cfDNA of pediatric solid tumor patients. One patient with metastatic osteosarcoma (case 1) at diagnosis demonstrated gains in chromosomes 1q, 5p, 6p, 10p, 19p, and 21 in the cfDNA sample (Fig. 3a) which was no longer present one month after completion of therapy (Fig. 3b). The same pattern of ctDNA abnormalities with an additional gain of chromosomes 7 and 8, was detected in all three follow-up samples collected between three and 12 months after therapy (Fig. 3c–e). At 12 months, the patient had a symptomatic recurrence at a distant bony site which was not evident on prior routine imaging surveillance. Following initiation of salvage chemotherapy, at 6 weeks, the abnormal clone was no longer detectable in the patient’s plasma (Fig. 3f). Thus, plasma-derived cfDNA analysis revealed the presence of recurrent disease nine months prior to clinical detection by conventional imaging techniques.

### LP-WGS of cfDNA in plasma may aid diagnosis and monitoring response to treatment of pediatric patients with solid tumors

**Fig. 4:**
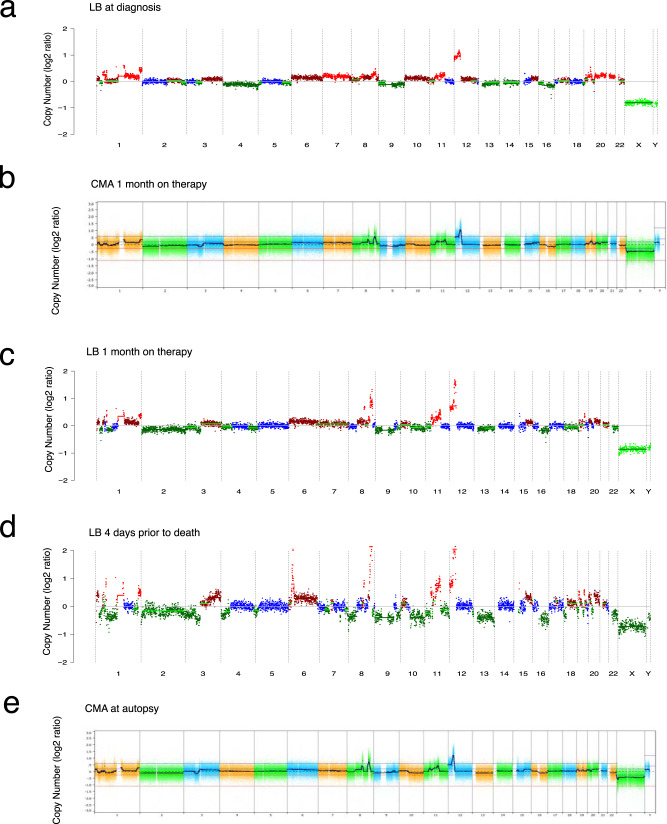
Treatment response monitoring using liquid biopsy in a germ cell tumor case. **a** CNA profile of ctDNA from plasma at diagnosis (patient 69) revealing complex abnormalities affecting almost all chromosomes with notable 12p gain. The calculated TFx was 16%. **b** Chromosomal microarray analysis of a pleural fluid sample with similar complex abnormalities and high-level gain of 12p. **c** CNA profile of plasma LB one month later, post cycle two of chemotherapy. The CNAs were detected for all chromosomes. The calculated TFx was 20%. **d** CNA profile of LB sample 3 months after diagnosis and one month before death. All CNAs were still detectable in the plasma of this patient with an additional high-level gain of 6p. The calculated TFx was 47%. **e** CMA plot at autopsy depicting highly complex CNAs affecting all chromosomes, but without the gain in 6p. For each serial sample, the estimated ichorCN log2 ratio is plotted on the y-axis, and chromosomes 1-22, X, and Y are shown on the x-axis. Red indicates CN gain, blue indicates CN neutral, and green indicates CN loss.

Patient 69 presented with a large mediastinal mass and large pleural effusion not amenable to biopsy due to the patient’s poor condition. A diagnosis of malignant germ cell tumor was made based on radiographic appearance and high alpha-fetoprotein (AFP) levels. The patient initially showed clinical improvement after initiating chemotherapy. However, after two cycles of standard chemotherapy, in the setting of decreasing AFP tumor markers, he had progression, and despite a change in systemic therapy, he died of disease. A germ cell tumor with somatic malignant transformation was suspected and confirmed at autopsy. LP-WGS of the plasma sample at diagnosis (Fig. 4a) prior to initiation of chemotherapy showed a highly complex pattern of whole chromosome and segmental gains and losses, including a gain of 12p and amplification of a region in 12q that included *KRAS*, alterations that are characteristic for germ cell tumor. After two cycles of chemotherapy, CMA analysis of a pleural fluid sample (Fig. 4b) and LP-WGS of plasma cfDNA (Fig. 4c) showed the same complex genetic abnormalities detected at diagnosis. The cfDNA sample (Fig. 4d) at three months on therapy and four days prior to the patient’s death as well as the CMA analysis of the tumor at autopsy (Fig. 4e), respectively, showed novel CNAs in addition to what had been detected in cfDNA at diagnosis.

Comparative LP-WGS of cfDNA (Supplemental Table 1) was performed for diagnostic and follow-up blood samples for 21 patients. In over half the patients (*n* = 12) there were no detectable CNAs in follow-up specimens, suggesting a response to treatment.

### Detection of ctDNA through fragmentomics analysis

The ability to detect somatic aberrations depends on the concentration of cfDNA fragments in the collected plasma which may vary by cancer type, stage of the disease, and disease burden. Recent literature has shown that the proportion of cfDNA fragments in the size range of 20–150 bp is correlated with cfDNA concentration in plasma as determined by mutation detection assays, and may be used to define high and low-burden tumor types using blood^12,32,33^. Hence, we sorted the diagnostic plasma samples by the median proportion of fragments less than 150 bp. We found that certain tumor types, including osteosarcoma and germ cell tumors, had the lowest median values overall compared to Ewing sarcoma and hepatic cancers, which had the highest values (Kruskal–Wallis *p* value = 0.023) (Supplemental Fig. 4a). Interestingly, we observed that ichorCNA derived tumor fractions and cfDNA concentration values were very similar to the short fragment proportion median ranks, reaffirming our categorization of low and high-tumor burden cancer types (Kruskal–Wallis *p* value = 0.61 and 0.013 respectively) (Supplemental Fig. 4b, c). Despite observing these discerning trends, more samples are necessary to make an affirmative conclusion.

### Mutation detection in plasma cfDNA

**Fig. 5:**
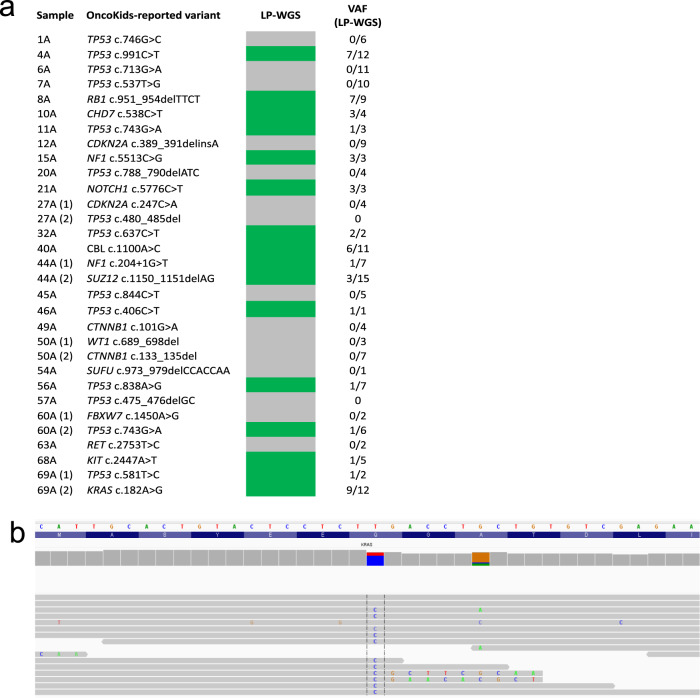
Mutation detection using low-pass whole genome sequencing data from liquid biopsies. **a** Summary of all variants identified with the OncoKids NGS-panel from the tumor that were inspected for presence in the matched LP-WGS data from the LB (*n* = 26 patients). (1) and (2) is used to refer to two different variants identified in the same patient. An indication of presence in LP-WGS is shown in green and an indication of absence is shown in gray. VAF is shown as a fraction of the number of reads for the variant allele over the total allele count. **b** IGV screenshot showing a *KRAS* c.182 A > G detection in LB from a germ cell tumor patient (wild-type allele = T, mutant allele = C).

In order to assess the sensitivity of our assay for detecting ctDNA from patients with pediatric solid tumors, we examined the LP-WGS data for somatic mutations. Targeted sequencing results were available from 58 primary or metastatic tumors profiled with our clinical targeted NGS-panel, OncoKids®^34^. Mutations in clinically significant cancer genes were detected in 26 patients (Fig. 5). We looked for the presence of these mutations in LP-WGS data from cfDNA samples using base counts generated from bam files and confirmed the presence of the variant allele by visual inspection using IGV. A total of 16 pathogenic or likely pathogenic mutations identified by OncoKids® were identified for 14 of our patients using the LP-WGS data (Fig. 5a). One germ cell tumor (patient 69), described above, was profiled with our OncoKids® NGS-panel and had a confirmed *KRAS* mutation, c.182 A > G (Fig. 5b). We were able to detect this mutation by LP-WGS in the plasma LB which revealed a similar variant allele frequency (VAF) as reported by OncoKids®, albeit at 4.4x depth of coverage. The VAF increased from 12 to 73% in the cfDNA during progression suggesting clonal expansion and evolution of the disease. This increase in VAF was also correlated with an increase in tumor purity estimates using ichorCNA, suggesting that mutations detected by LP-WGS could provide an alternative approach for monitoring clonal dynamics. Similarly, the tumor sample from a patient with metastatic osteosarcoma (patient 11) was positive for a *TP53* missense (c.743 G > A) variant of strong clinical significance, at a VAF of 28%. Mutational analysis of LP-WGS data allowed us to detect this same mutation at a VAF of 33%. *TP53* and *KRAS* were among the most mutated genes identified from both the LP-WGS ctDNA data and OncoKids® tumor data across all pediatric solid tumor cases. Further studies are required to determine if this approach will be clinically feasible on a prospective basis, at least for some patients, or whether a companion targeted capture NGS-based panel approach will be required.

### Detection of translocations by hybridization-based capture and sequencing of cfDNA

Chromosomal translocations contribute to tumorigenesis in many pediatric cancers. To better understand the clinical applicability of cfDNA assays, we developed a hybridization capture-based panel targeting the most common translocation partners in two pediatric cancers, specifically *EWSR1* for Ewing sarcoma and *FOXO1* in ARMS^5^. We targeted the most common breakpoints in these tumors, spanning exons 8–11 and introns 7–12 within *EWSR1*, and intron 1 within *FOXO1*^5^. We evaluated the panel using tumor DNA samples from five patients (four with *EWSR1* fusions and one with a *FOXO1* fusion, as previously identified by OncoKids®^34^). We detected *EWSR1* fusions in three patients with Ewing sarcoma as well as a *FOXO1* fusion in a patient with rhabdomyosarcoma, confirming the specificity of our assay. One tumor sample with a confirmed *EWSR1* fusion by OncoKids® was not detected with our assay in the primary tumor. However, the fusion was detected with the same panel using cfDNA, suggesting that intra-tumor heterogeneity in the primary tumor could be a possible reason for not detecting this fusion. We then tested 19 cfDNA samples from 17 patients, including 12 with identified *EWSR1* fusions, two with identified *FOXO1* fusions by OncoKids®^34^, and three negative controls. We were able to identify *EWSR1* fusions in 10 of the 12 patients with Ewing sarcoma (Supplemental Fig. 5a), including two patients who had a CNA-negative LP-WGS profile at diagnosis, one with localized (patient 28) and one with metastatic disease (patient 26), (Supplemental Fig. 5b, c). *FOXO1* fusions were identified in the plasma of both patients with ARMS, one of which was negative for CNAs by LP-WGS (patient 38). The controls were negative for *EWSR1* and *FOXO1* fusions. Combined, these data suggest that the detection of gene fusions using cfDNA is possible in patients with pediatric solid tumors.

## Discussion

Establishing the clinical applicability of plasma-based liquid biopsies in pediatric cancer patients versus adult patients has been challenging due to the limited sample volumes from infants and young children, small cohort sizes for individual tumor types, as well as the varied nature of the genomic alterations that characterize pediatric solid tumors. Nevertheless, several studies have described the feasibility of NGS-based approaches in pediatric solid tumors^19,21–24,30,31,35–37^. For example, to detect neuroblastoma-specific markers for LB biobanking strategies in 84 infants, Lodrini et al. showed that as little as 1–2 ml of blood plasma, CSF, or urine had sufficient cfDNA for disease monitoring and ultimately clinical implementation of LB assays^23^. However, whether this was a disease-specific finding, and whether cfDNA yield is correlated with location, histology, or tumor burden has yet to be determined. Shulman et al. recently reported that ctDNA can be detected in plasma samples from ~50% of pediatric Ewing sarcoma and osteosarcoma patients^35^ and Shah et al.^29^ successfully detected *EWSR1* or *FOXO1* fusions in patients with Ewing sarcoma or ARMS, respectively. However, similar to MAPPYACTS^20^, it is worth noting that many of the large cohort studies included patients with recurrent and/or metastatic disease in which the probability of detecting ctDNA in serum may be increased compared to patients with primary and/or localized disease.

We previously reported the development of an LB assay for retinoblastoma using as little as 100 microliters of the aqueous humor of the eye and ultra-low-pass WGS to detect *RB1* mutations and RB-associated CNAs^25–27^. Our goal for the present study was to determine whether plasma-based LB and LP-WGS could be used to aid in clinical diagnosis and prognosis of pediatric solid tumors in general, to monitor response to therapy, and to demonstrate early evidence for relapse.

In this study, we demonstrated high sensitivity in detecting ctDNA across different pediatric solid tumor types. The overall CNA detection rate by LP-WGS was ~70% (26 of 37 patients) when considering only those patients with no systemic therapy or surgery prior to enrollment. Notably, 18 of 27 (67%) patients enrolled at diagnosis with localized disease had abnormal copy number profiles. To our knowledge, this is one of the few studies to show the applicability of ctDNA detection in pediatric solid tumor patients with localized disease. Moreover, even with prior resection of the primary tumor or chemotherapy, patients enrolled at diagnosis may still have sufficient ctDNA in their plasma to be detected by LP-WGS.

The majority of osteosarcoma patients profiled here had localized disease and detectable CNAs in cfDNA at diagnosis. The absence of CNAs in follow-up samples was consistent with clinical response to therapy. For example, as shown in Fig. 4, patient one was diagnosed with metastatic osteosarcoma, and had a negative LP-WGS CN profile one month off therapy, consistent with a molecular response to treatment. However, he had CNAs in the plasma as early as three months after the completion of therapy, and nine months prior to clinical detection of recurrence in a distant bony site. Our data thus suggest that serial LB of patients may provide a sensitive means for detecting early response and relapse.

The potential application of this approach as an aid in the primary diagnosis of patients is illustrated in patient 69 with a germ cell tumor. Three available LP-WGS profiles from an LB of the pleural fluid and plasma-derived ctDNA showed a complex degree of genetic abnormalities involving most of the chromosomes, but which included copy number gains of chromosome 1 and 12 that are characteristic of germ cell tumor. The use of LP-WGS of cfDNA in conjunction with other clinical biomarkers such as AFP, may ultimately provide a non-invasive means of molecular diagnosis and evaluating clonal evolution in pediatric patients with solid tumors.

To improve sensitivity in detecting ctDNA, fragmentomic analysis is an effective method for distinguishing ctDNA from non-cancer-derived cfDNA fragments in different tumor types such as glioma^32,33^, renal and pancreatic cancer^32^, as well as soft-tissue sarcoma^28^. By looking at the proportion of short fragments <150 bp we show that pediatric tumor types may also be classified by tumor burden (Supplemental Fig. 4). Hepatic tumors and Ewing sarcoma appear to have the highest tumor burden with a high proportion of short ctDNA-derived fragments less than 150 bp. Similar patterns were evident when comparing tumor fraction estimates from ichorCNA^30^ and cfDNA yield per mL between the different cancer types. Combined, we show that ctDNA detectability may vary by tumor type, since some types of cancer may carry a higher proportion of ctDNA fragments. This hypothesis has been tested recently by a quantitative approach to measuring mutant ctDNA fragments in adult cancer patients^12^. Neuroblastoma and prostate cancers seemed to have the highest proportion of *KRAS* mutant ctDNA fragments when compared to medulloblastomas and gliomas^12^. Since our analysis was limited by the small cohort size and varying patterns between tumor types, a larger cohort would allow us to affirm our conclusions on the applicability of fragment size analysis in detecting ctDNA and distinguishing tumor types in pediatric cancers.

In a proof-of-concept retrospective analysis, we examined the LP-WGS data for 26 patients in whom clinically significant mutations had been identified by our clinical NGS-based OncoKids® assay of the tumor tissue. We identified 16 hotspot mutations (in 14 patients) in a variety of oncogenes and tumor suppressor genes using cfDNA, including *PIK3CA, TP53, and CTNNB1*, demonstrating that our LP-WGS assay was sensitive in detecting the low but significant number of mutations present in pediatric solid tumors^17^. This approach, however, may be limited by the ctDNA fraction in plasma and the low sequencing depth using LP-WGS. Moreover, our fusion capture panel was able to detect fusions with high precision (12 of 14) in Ewing sarcoma and ARMS using cfDNA samples (Supplemental Fig. 5). Two Ewing sarcoma cases were negative for fusions in plasma and had no detectable ctDNA by LP-WGS, suggesting a low prevalence of circulating tumor as the likely explanation for these negative results. Additionally, three of the fusion-positive patients, two with an *EWSR1* and one with a *FOXO1* fusion, had negative CNA profiles by LP-WGS underscoring the importance of comprehensive characterization of aberrations by both a targeted panel and LP-WGS. It is worth noting that we were limited by sample size for RMS since this type of tumor and *FOXO1* translocations are rare^5^. Targeted NGS panels with a higher depth of coverage are in development and are likely to have a higher sensitivity for identifying the most common gene mutations and fusions in both CNS and pediatric solid tumors.

Determination of the clinical applicability of LB assays such as the ones described in the present study will require validating their use in larger cohorts of patients with pediatric solid tumors. Serial studies using NGS-based LB assays will lead to a greater understanding of how they can be employed to monitor patients from diagnosis through treatment and recurrence.

## Methods

### Patient sample collection and processing

Peripheral blood (1–10 ml) was collected in EDTA tubes and processed within a median of 1 h and 30 min by centrifugation at 2000 × *g* for 10 min to separate plasma and a buffy coat. The supernatant was removed, and a second centrifugation step was performed at 16,000 × *g* for 10 min to remove cell debris. All centrifugation steps were performed at 4 °C. The plasma was frozen until DNA isolation. cfDNA was extracted from 500 ul to 9 ml (median 3 ml) plasma samples using the MagMAX™ Cell-Free Total Nucleic Acid Isolation Kit according to the manufacturer’s instructions (Thermo Fisher Scientific, Waltham, MA). The abundance and quality of cfDNA in the extracted samples were assessed using a bioanalyzer Cell-free DNA ScreenTape analysis (Agilent, Santa Clara, CA) and Quantus™ Fluorometer (Promega, Madison, WI).

### Library preparation and sequencing

Whole genome sequencing libraries were constructed with the xGen Prism DNA Library Prep Kit according to the manufacturer’s instructions and applying 13 cycles of PCR with the addition of fixed single-stranded Unique Dual Molecular Identifier indexes (UDMIs) (Integrated DNA Technologies, Coralville, IA) using 5 ng of cfDNA input. All libraries were paired-end sequenced on an Illumina NextSeq 500 or an Illumina HiSeq 4000 (San Diego, CA) at an average of 4.4x depth of coverage, which is deemed low coverage.

### Copy number alteration and tumor fraction analysis

Reads were aligned to the 1000 genomes phase 2 reference genome (hs37d5) which includes build GRCh37 and decoy sequences: (ftp://ftp-trace.ncbi.nih.gov/1000genomes/ftp/technical/reference/phase2_reference_assembly_sequence/hs37d5.fa.gz) using the Illumina Dragen 3.7.3 aligner. The ichorCNA algorithm was subsequently applied using the depth of coverage obtained from 500 kb bins across the genome^38^. When available, LP-WGS data from the LB samples were compared with CNA profiles of the matching primary tumors generated from CMA assays (CytoScanHD or OncoScan, Thermo Fisher Scientific, Waltham, MA). Briefly, all 59 Cytoscan or Oncoscan arrays performed on the tumor tissue were processed using ASCAT and NxClinical (BioDiscovery, El Segundo, CA) using the default settings, except that the Piecewise Constant Fitting (PCF) penalty was increased to 95 to account for FFPE degradation of samples. Subsequently, the segmented calls from LP-WGS and CMA were binned into 1 Mb regions across the entire genome. Pearson’s correlation for each pair of samples across all bins over autosomes was calculated for all CMA samples and LP-WGS samples with purity greater than 10%. All samples with a Pearson’s correlation coefficient (*r*^2^) less than 0.7 were manually examined.

### Fragment size, tumor concentration, and fraction analysis

Insert size distributions across the entire genome were calculated using Illumina Dragen 3.7.3. The ratio of the number of fragments with sizes less than or equal to 150 bp and greater than 150 bp but less than 500 bp were calculated for all samples. Tumor fraction was estimated for all samples using the ichorCNA variant calling pipeline as previously described in ref. ^38^. Briefly, the algorithm uses a Hidden Markov Model (HMM) to predict copy number segments and to estimate the circulating tumor content from total cfDNA sequenced. The details of this approach have been previously described in ref. ^38^. For each of the categories (i.e., proportion of fragments <150 bp, tumor fraction, and tumor concentration) samples were classified by tumor type, and a Kruskal–Wallis test was performed between cancer types. Only samples collected at the time of diagnosis were used for this analysis. A *p* value <0.01 was considered statistically significant.

### Mutational analysis and targeted sequencing for fusion detection

Targeted sequencing results from primary or metastatic tumors profiled with our clinical NGS-panel, OncoKids®, were reviewed for 26 cases^34^. The LP-WGS data from the LB samples were assessed to determine whether the same mutations could be detected. For mutations identified by OncoKids®, we used GetBaseCountsMultisample, to verify the presence of a mutation in LP-WGS (https://github.com/mskcc/GetBaseCountsMultiSample). We further verified the presence of mutations by LP-WGS using Integrative Genomics Viewer (IGV)^39^.

For detection of the *EWSR1* and *FOXO1* fusions in Ewing sarcoma and ARMS patients, respectively, we designed a custom panel to test in tumor DNA using a hybridization-based capture method (Twist Bioscience, South San Francisco, CA). Since UMIs only help with correcting PCR and chemistry artifacts when detecting point mutations, we trimmed the UMIs using the Dragen 3.7.3 aligner and realigned the reads to build 37 of the human reference genome. This increased the sensitivity to detect fusions. We used Illumina Manta (version 1.6.0) in the “targeted” mode (https://github.com/Illumina/manta) to detect structural variants. All statistical analysis was carried out using R version 4.0.4.

### Reporting summary

Further information on research design is available in the Nature Research Reporting Summary linked to this article.

## Supplementary information

REPORTING SUMMARY

Supplemental material

## Data Availability

The ability to share primary sequence data were not included in the informed consent signed by patients. A data use agreement is required to access the data. Sequencing data from patients who are deceased have been deposited in EGAS00001006913.

## Code availability

We used publicly available packages using R version 4.0.3 for our analysis. The code used to generate figures is available upon request.
