# Supplementary material for: Combined low-pass whole genome and targeted sequencing in liquid biopsies for pediatric solid tumors
Source: NPJ Precis Oncol. 2023 Feb 20;7:21. doi: 10.1038/s41698-023-00357-0 (PMC9941464; doi:10.1038/s41698-023-00357-0)
Supplement: Supplementary file 2 — Supplemental material [file 41698_2023_357_MOESM2_ESM.pdf]

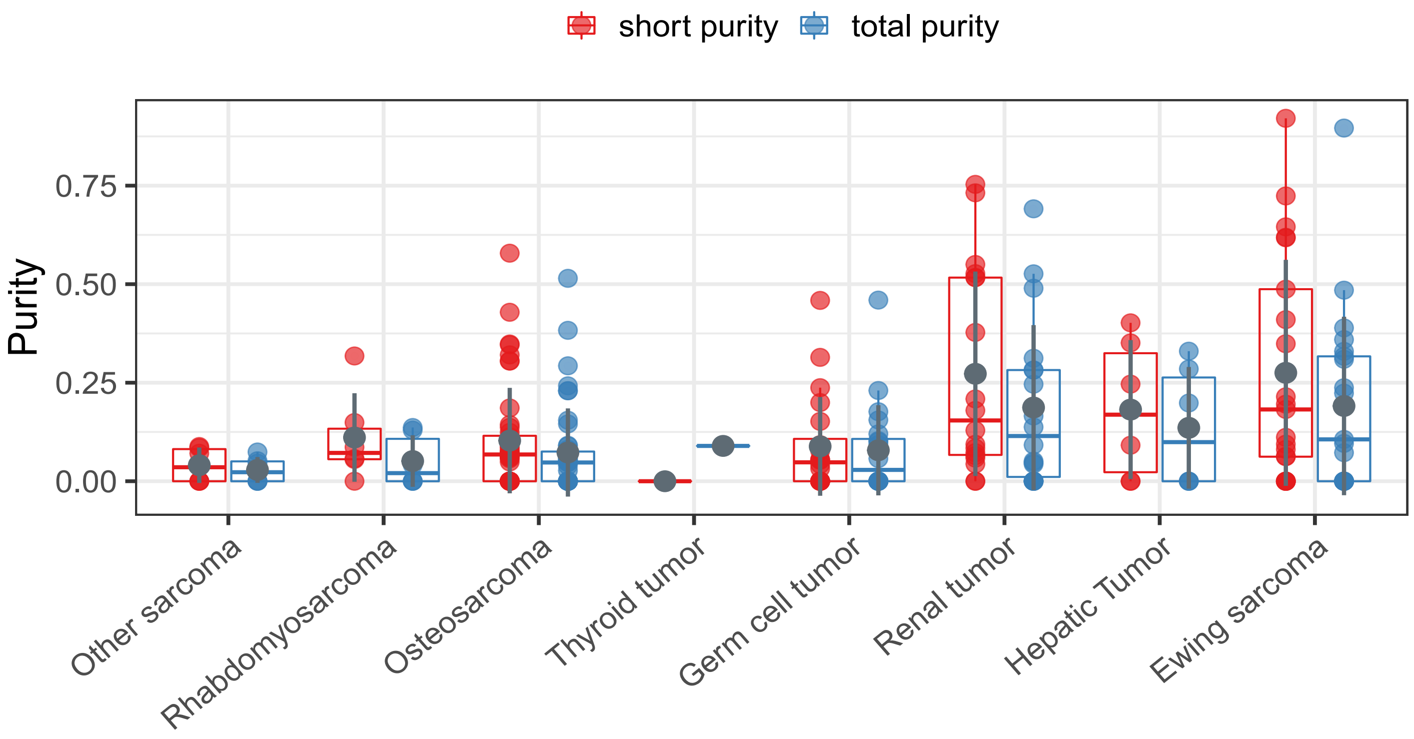

Supplemental Figure 1 Tumor fraction estimates with and without in silico size selection of reads from 90-150bp. Boxplots of tumor fraction (purity) estimated using ichorCNA are shown for the same samples with in-silico size selection of fragments between 90-150bp (red-short purity) and without in silico size selection (blue-total purity). Each point represents a different sample. Error bars (gray) show the mean and standard deviation of tumor purity for each group. Center line (red or blue) shows the median and bounds of box and whiskers show the range of tumor purity.

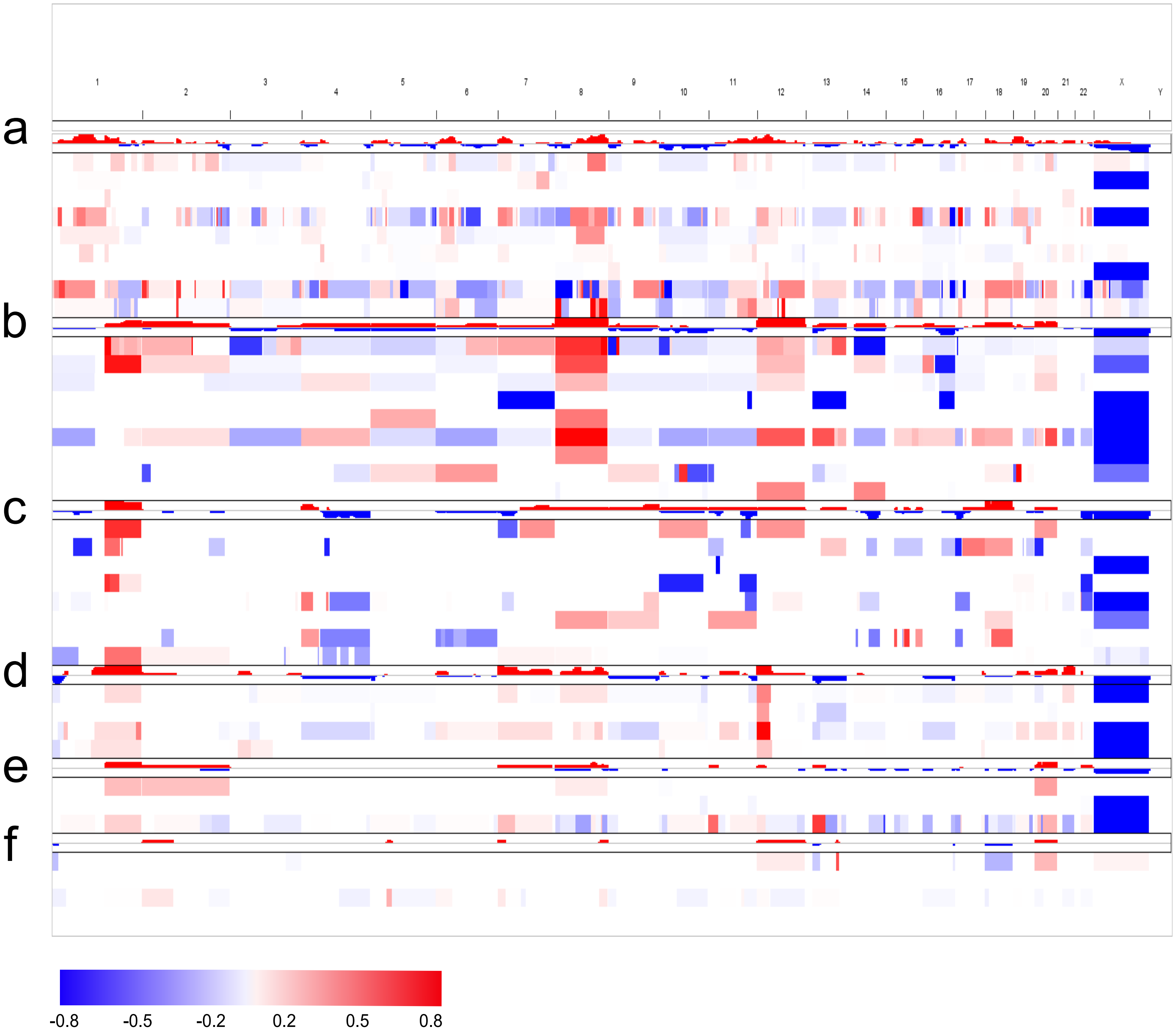

Supplemental Figure 2 Copy number alteration detection per tumor type in pediatric solid tumors at diagnosis or relapse a) Osteosarcoma (n=9) **b)** Ewing sarcoma (n=9) **c)** Renal tumor (n=8) **d)** Germ-cell tumor (n=4) **e)** Hepatic tumor (n=3) **f)** Other sarcomas (n=3). Thyroid tumors are not shown since there were no CNA positive cases. Each row represents a CNA positive case either at diagnosis or relapse, and each column represents a chromosome. 1-22, X, Y. The top panel for each tumor type represents the summary of CNAs across all chromosomes. Red: copy number gain, Blue: copy number loss (see scale bar).

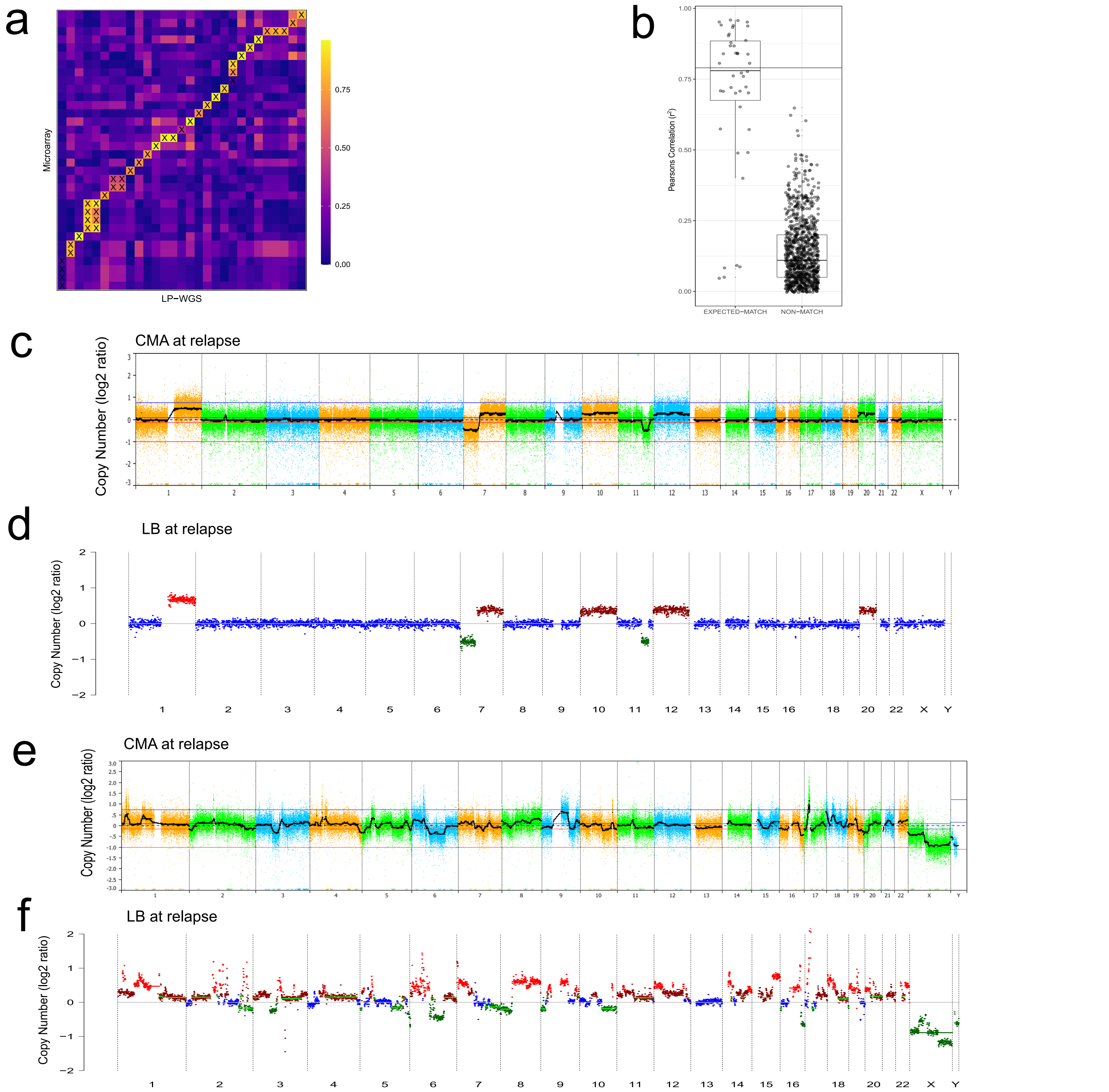

Supplemental Figure 3 Correlation of copy number changes identified by chromosomal microarray analysis of tumor tissue and low-pass whole-genome sequencing of plasma samples a) Heatmap of correlation scores (r^2) for CNA detected from Low-Pass Whole Genome Sequencing (LP-WGS) (n=29) data having tumor fractions of at least 0.1 and Cytoscan or Oncoscan microarrays (n=34) from the tumor tissue. The segmented calls were binned into 1Mb regions across the entire genome and Pearson's correlations for all bins for each pair of samples are calculated. The correlation of matched LB and tumor samples are shown by an X. **b)** Boxplot of Pearson's correlation for expected match and non-match pair of samples across all bins for all CMA samples and LP-WGS samples with ichorCNA purity greater than 0.1. A horizontal line indicates the median proportion for each cancer type and bounds show the range. Example of a Wilms tumor **c)** Oncoscan profile (CMA) **d)** LP-WGS profile with a Pearson's correlation r^2 of 0.95. The calculated tumor fraction (TFx) from LP-WGS was 59%. Tumor fraction is indicative of ichor purity estimate. Example of an osteosarcoma case profile **e)** Oncoscan profile (CMA) **f)** LP-WGS profile with a Pearson's correlation r^2 of 0.5. The calculated TFx from LPWGS data was 37%. The estimated CN log_2 ratio is plotted on the y-axis against chromosome numbers 1-22, X, and Y. Oncoscan panel: Deviation above 0 represents CN gain and deviation below 0 represents CN loss. LP-WGS-panel: Red indicates CN gain, Blue indicates CN neutral and green indicates CN loss. X-axis represents chromosomal numbers, 1-22, X, and Y.

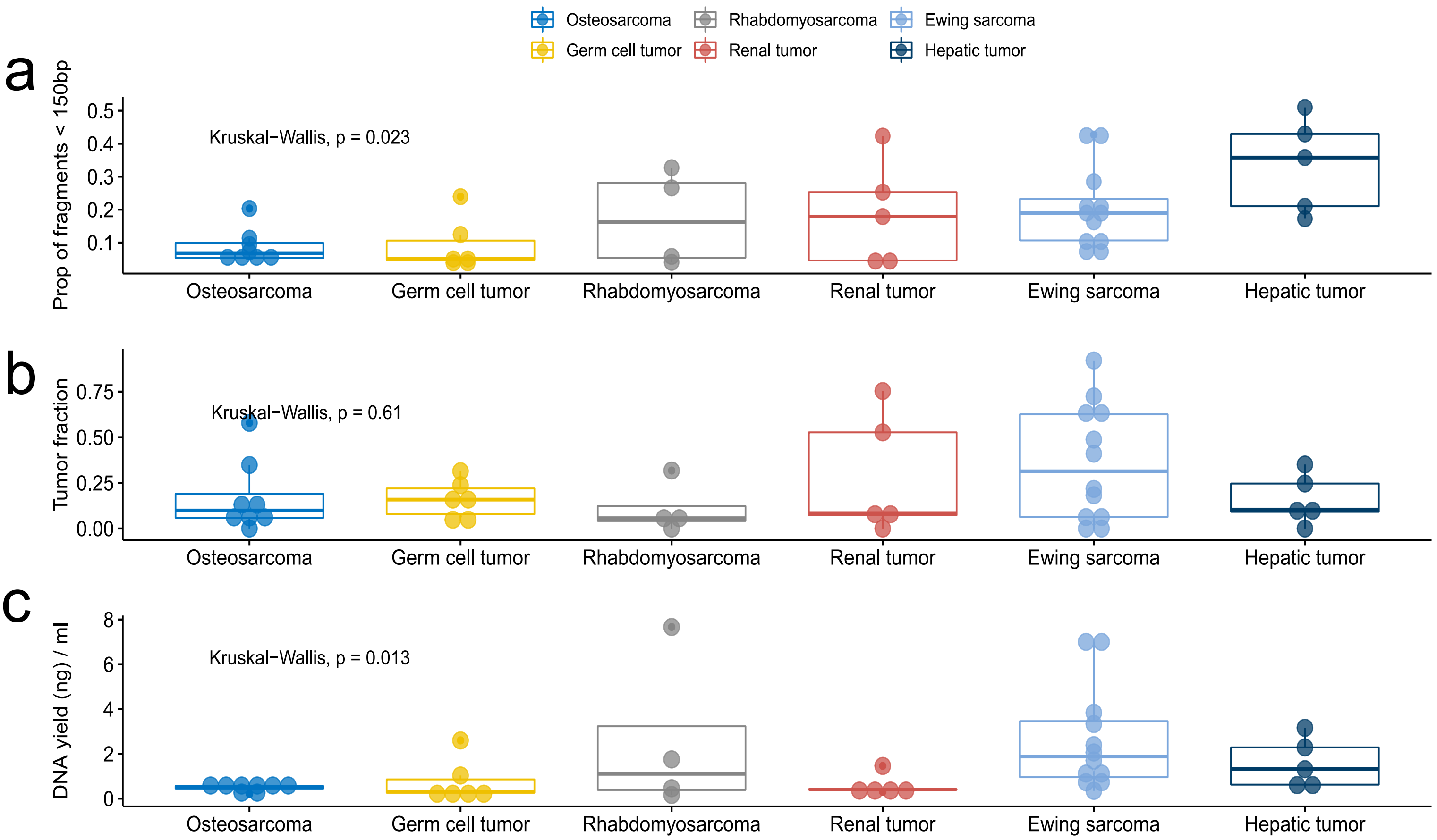

Supplemental Figure 4 Pan-cancer categorization of high and low tumor burden types using liquid biopsies collected at the time of diagnosis. Boxplots by each tumor type having at least three liquid biopsy samples collected at baseline showed **a)** Proportion of cfDNA fragments below 150bp in different pediatric tumor types. Samples are median ranked by proportion of fragments <150bp. The Kruskal-Wallis test indicated a significant difference in the median fragment sizes among tumor types (p=0.023). **b)** Tumor fraction estimated by ichorCNA. The Kruskal-Wallis test indicated a non significant (p=0.61) association of tumor fraction among different tumor types. **c)** cfDNA yield (ng/ml) among different pediatric tumor types. The Kruskal-Wallis test indicated a significant (p=0.0013) association of cfDNA yield among different tumor types. The horizontal line indicates the median value for each cancer type and bounds of boxplot show the range.

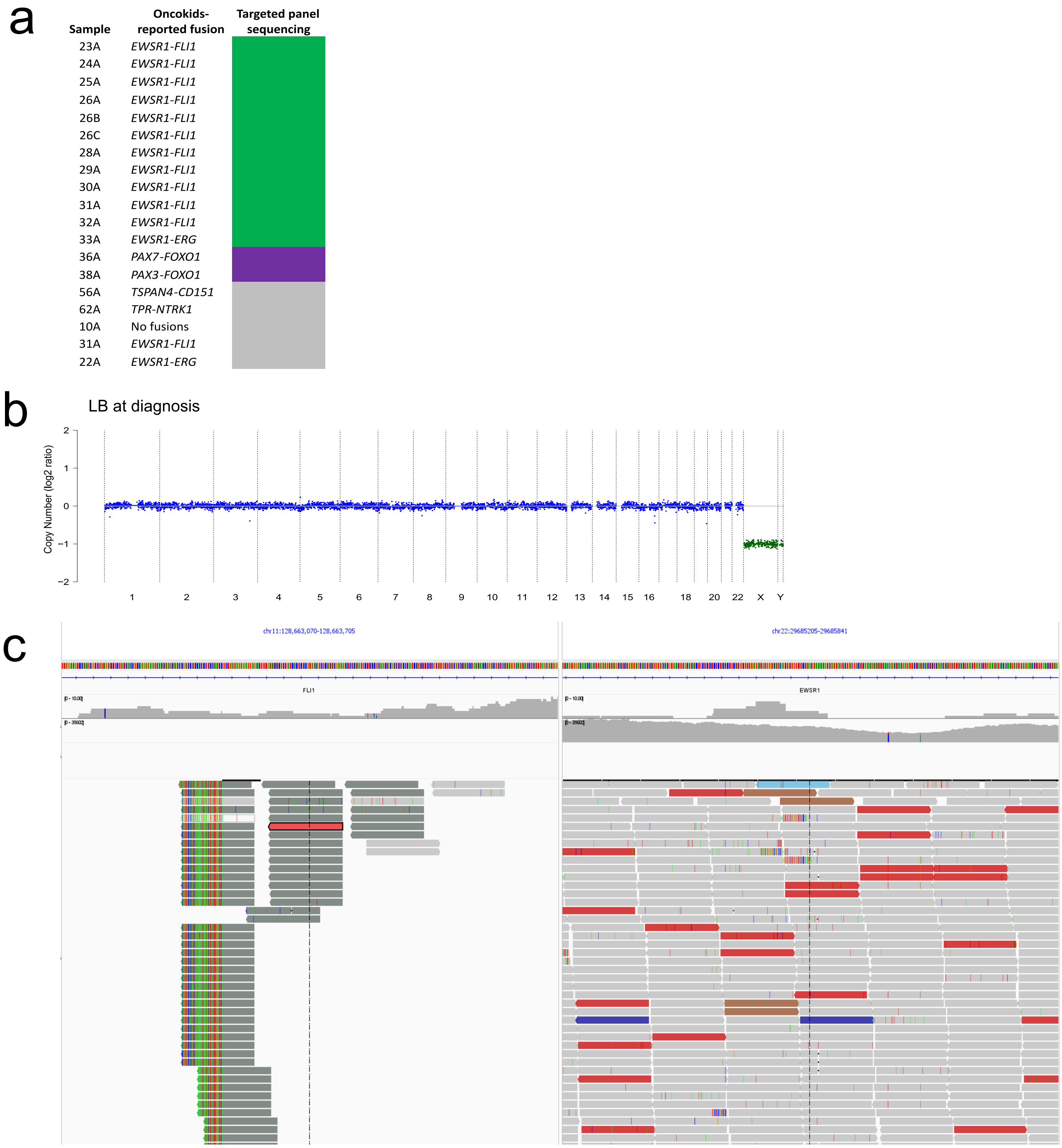

Supplemental Figure 5 Targeted hybrid fusion panel development detects translocations in cfDNA from patients with pediatric solid tumors a) Summary of all Oncokids-identified translocation events from the tumor tissue that were included in the hybrid fusion capture panel for detection in cfDNA (n=19 samples, patient 26 had three available samples). Indication of translocation presence in cfDNA is shown by green color for *EWSR1* and purple for *FOXO1*. An indication of absence is shown by a gray color **b)** CN plot of an Ewing sarcoma patient (patient 26) with localized disease at baseline. The patient was male. No CN events were detected in cfDNA. LP-WGS-panel: Red indicates CN gain, Blue indicates CN neutral, and green indicates CN loss. X-axis represents chromosomal numbers, 1-22, X, and Y. **c)** IGV screenshot showing an *EWSR1-FLI1* translocation event in cfDNA from patient 26. The region displayed on the IGV plot is a capture from the genomic location chr11:128663277 (*FLI1* genomic). The gray reads indicate a translocation event that maps to chr22:29685700 (*EWSR1* genomic). Soft-clip bases (>40), that do not match the chr11:128663277 sequence, are shown as colorful boxes on the left of gray reads and map to chr22:29685435-29685481 (*EWSR1* genomic).

Patient	Tumor type	Initial diagnosis /Relapse at Study Enrollment	Stage at Diagnosis/ Type of Relapse	Site(s) of disease	Sex/Age (yrs)	Timepoint	CMA available (tumor)	CMA result (tumor)	LPWGS result (plasma)	Oncokids result (tumor)	Mutation detection by LP-WGS (plasma)	Fusion detection (plasma)
1A	Osteosarcoma	Initial diagnosis	Metastatic	R proximal femur	M/15	At diagnosis	YES	Abnormal	Abnormal	*TP53* c.746G>C, p.Arg249Thr VAF 58%	Not detected	
1B						1 mo off therapy			Non-informative			
1C						3 mo off therapy - no evidence of disease			Abnormal			
1D						6 mo off therapy - no evidence of disease			Abnormal			
1E			Distant	L proximal femur		At relapse			Abnormal			
1F						Pre surgery			Non-informative			
1G						End of therapy			Non-informative			
2A	Osteosarcoma	Initial diagnosis	Localized	L proximal femur	M/11	At diagnosis	YES	Abnormal	Abnormal	No clinically established variants		
2B						Post therapy			Abnormal			
2C						Post surgery			Non-informative			
2D						End of therapy			Non-informative			
2E			Distant	L lung nodules		At relapse			Non-informative			
2F						End of therapy			Non-informative			
2G						9 mo off therapy - no evidence of disease			Non-informative			
3A	Osteosarcoma	Initial diagnosis	Localized	R distal femur	F/11	At diagnosis	YES	Abnormal	Abnormal	*IGF1R* gene amplification		
4A	Osteosarcoma	Initial diagnosis	Metastatic	R proximal tibia	F/7	At diagnosis	YES	Abnormal	Abnormal	*TP53* c.991C>T, p.Gln331Ter VAF 83%	*TP53* c.991C>T VAF 58%	
4B						7 mo off therapy			Non-informative			
4C			Distant	R lung nodules		At relapse			Non-informative			
5A	Osteosarcoma	Initial diagnosis	Localized	R distal femur	M/12	At diagnosis	YES	Abnormal	Non-informative	No clinically established variants		
6A	Osteosarcoma	Initial diagnosis	Localized	R proximal humerus	F/11	At diagnosis-during therapy	YES	Abnormal	Non-informative	*TP53* c.713G>A, p.Cys238Tyr VAF 33%	Not detected	
7A	Osteosarcoma	Initial diagnosis	Localized	R proximal tibia	M/19	At diagnosis	YES	Abnormal	Abnormal	*TP53* c.537T>G, p.His179Gln VAF 66%	Not detected	
8A	Osteosarcoma	Initial diagnosis	Localized	L proximal humerus	F/12	At diagnosis	YES	Abnormal	Abnormal	*RB1* c.951_954delTTCT, p.Ser318Asnfs*13 VAF 91%	*RB1* c.951_954delTTCT VAF 78%	
9A	Osteosarcoma	Initial diagnosis	Localized	L distal tibia	F/15	At diagnosis	YES	Non-informative	Non-informative	No clinically established variants		
10A	Osteosarcoma	Relapse	Distant	Bilateral lung nodules	M/17	At relapse-during therapy	YES	Abnormal	Abnormal	*CHD7* c.538C>T, p.Arg195Cys VAF 22%	*CHD7* c.538C>T VAF 75%	Not detected
11A	Osteosarcoma	Relapse	Distant	R lung metastases	F/10	At relapse	YES	Abnormal	Non-informative	*TP53* c.743G>A, p.Arg248Gln VAF 28%, *MYC* amplification	*TP53* c.743G>A VAF 33%	
12A	Osteosarcoma	Relapse	Distant	L bone (glenoid) mass	F/15	At relapse	YES	Abnormal	Abnormal	*CDKN2A* c.389_391delinsA, p.Leu130Glnfs*11 VAF 60%	Not detected	
12B						3 mo off therapy - enhancing lung lesion			Non-informative			
12C			Distant			19 mo off therapy - enhancing lung lesion			Non-informative			
13A	Osteosarcoma	Relapse	Distant	R lung nodule	F/18	At relapse	NO	NA	Non-informative	No OncoKids report		
14A	Osteosarcoma	Relapse	Distant	L leg mass - recurrent osteosarcoma	M/20	At relapse	NO	NA	Non-informative	No OncoKids report		
14B						Post therapy			Non-informative			
15A	Osteosarcoma	Relapse	Distant	Increased uptake right proximal humerus	M/13	At relapse	YES	Non-informative	Non-informative	*NF1* c.5513C>G, p.Ser1838Cys VAF 49%	*NF1* c.5513C>G VAF 100%	
16A	Osteosarcoma	Relapse	Distant	Bilateral lung metastases	M/15	At relapse-during therapy	NO	NA	Non-informative	No OncoKids report		
17A	Osteosarcoma	Relapse	Distant	Bilateral enlarging lung metastases	M/19	At relapse	YES	Abnormal	Non-informative	No OncoKids report		

18A	Osteosarcoma	Relapse	Distant	3rd relapse - L clavical	M/19	At relapse	YES	Abnormal	Abnormal	No OncoKids report		
18B						4 mo off therapy - no evidence of disease			Non-informative			
18C						12 mo off therapy - no evidence of disease			Non-informative			
18D						15 mo off therapy - no evidence of disease			Non-informative			
19A	Osteosarcoma	Relapse	Distant	L distal femur and proximal tibia	F/11	At relapse	YES	Abnormal	Abnormal	Gene amplification of *CDK4* and *GLI1*		
19B				Bilateral lung metastases		At relapse			Abnormal			
19C						Post radiation			Abnormal			
19D						Post therapy			Abnormal			
20A	Osteosarcoma	Relapse	Distant	Bilateral lung metastases	F/18	At relapse	YES	Abnormal	Non-informative	*TP53* c.788_790delATC, p.Asn263fs VAF 30%	Not detected	
21A	Ewing sarcoma	Initial diagnosis	Localized	L distal femur	F/14	At diagnosis	YES	Non-informative	Non-informative	Abnormal *CBFB-MYH11* fusion; *NOTCH1* c.5776C>T, p.Arg1926Cys VAF 50%	*NOTCH1* c.5776C>T VAF 100%	
22A	Ewing sarcoma	Initial diagnosis	Metastatic	Hemithorax mass	F/6m	At diagnosis-during therapy	YES	Non-informative	Non-informative	*EWSR1-ERG* fusion		Not detected
23A	Ewing sarcoma	Initial diagnosis	Localized	R 4th rib	M/7	At diagnosis	YES	Abnormal	Abnormal	*EWSR1-FLI1* fusion		Detected
24A	Ewing sarcoma	Initial diagnosis	Localized	R 12th rib	M/11	At diagnosis	YES	Abnormal	Abnormal	*EWSR1-FLI1* fusion		Detected
25A	Ewing sarcoma	Initial diagnosis	Localized	R proximal femur	F/13	At diagnosis	YES	Abnormal	Abnormal	*EWSR1-FLI1* fusion		Detected
26A	Ewing sarcoma	Initial diagnosis	Metastatic	L distal femur	M/10	At diagnosis	YES	Abnormal	Non-informative	*EWSR1-FLI1* fusion		Detected
26B				Bilateral lung metastases, L2 & L3 vertebral bodies, 7th rib		At relapse			Abnormal			Detected
26C						Refractory disease			Abnormal			Detected
27A	Ewing sarcoma	Initial diagnosis	Metastatic	R proximal humerus	F/19	At diagnosis	YES	Abnormal	Abnormal	*CDKN2A* c.247C>A, p.His83Asn VAF 50%, *TP53* c.480_485del, p.Met160_Ala161del 50%	Not detected	
27B						Post therapy			Non-informative			
27C						Pre surgery			Non-informative			
27D						Post therapy			Non-informative			
27E						Post therapy			Non-informative			
27F						Post radiation			Non-informative			
28A	Ewing sarcoma	Initial diagnosis	Localized	R buttock	F/17	At diagnosis	YES	Non-informative	Non-informative	*EWSR1-FLI1* fusion		Detected
29A	Ewing sarcoma	Initial diagnosis	Metastatic	L calf	F/12	At diagnosis	YES	Abnormal	Abnormal	*EWSR1-FLI1* fusion		Detected
30A	Ewing sarcoma	Initial diagnosis	Localized	R ilium	F/6	At diagnosis	NO	NA	Abnormal	*EWSR1-FLI1* fusion		Detected
31A	Ewing sarcoma	Initial diagnosis	Localized	Frontal bone of skull	F/14	At diagnosis	YES	Non-informative	Non-informative	*EWSR1-FLI1* fusion		Not detected
32A	Ewing sarcoma	Initial diagnosis	Metastatic	L proximal femur	M/19	At diagnosis	NO	NA	Abnormal	*EWSR1-FLI1* fusion and *TP53* c.637C>T, p.Arg213* VAF 81%	*TP53* c.637C>T VAF 100%	Detected
33A	Ewing sarcoma	Initial diagnosis	Metastatic	Sternum	M/17	At diagnosis	YES	Abnormal	Abnormal	*EWSR1-ERG* fusion		Detected
34A	Ewing sarcoma	Relapse	Distant	Multiple sites	F/28	At relapse	NO	NA	Abnormal			
35A	Ewing sarcoma	Relapse	Distant	R gluteal	M/21	At relapse-during therapy	YES	Abnormal	Abnormal	*EWSR1-FLI1* fusion		Detected
36A	Alveolar rhabdomyosarcoma	Initial diagnosis	Localized		F/4	At diagnosis	YES	Abnormal	Abnormal	*PAX7-FOXO1* fusion		Detected
37A	Embryonal rhabdomyosarcoma	Initial diagnosis	Localized		M/7	At diagnosis	YES	Abnormal	Non-informative	No clinically established variants		
38A	Alveolar rhabdomyosarcoma	Initial diagnosis	Localized		F/14	At diagnosis-post surgery	YES	Abnormal	Non-informative	*PAX3-FOXO1* fusion		Detected
39A	Alveolar rhabdomyosarcoma	Initial diagnosis	Metastatic		F/12	At diagnosis	YES	Abnormal	Non-informative	*PAX3-FOXO1* fusion. Amplifications of *CDK4*, *MYCN*, and *GLI1* genes		
39B						Post therapy			Abnormal			

40A	Embryonal rhabdomyosarcoma	Relapse	Local	Proximal sigmoid colon and omentum	M/4	At relapse-during therapy	YES	Abnormal	Non-informative	*CBL* c.1100A>C ,p.Gln367Pro VAF 98%	*CBL* c.1100A>C VAF 55%	
41A	Synovial sarcoma	Initial diagnosis	Localized		F/13	At diagnosis	YES	Abnormal	Abnormal	*SS18-SSX1* fusion		
42A	Synovial sarcoma	Relapse	Distant	Bilateral lung metastases	M/11	At relapse	YES	Abnormal	Non-informative	*SS18-SSX2* fusion		
43A	Malignant peripheral nerve sheath tumor	Initial diagnosis	Localized	L paraspinal	F/11	At diagnosis	YES	Non-informative	Non-informative	*BCOR-CCNB3* fusion		
44A	Malignant peripheral nerve sheath tumor	Initial diagnosis	Localized	Retroperitoneum	F/15	At diagnosis	YES	Abnormal	Abnormal	*NF1* c.204+1G>T p.? VAF 98%, *SUZ12* c.1150_1151delAG, p.Leu385Profs*10 VAF 96%	*NF1* c.204+1G>T VAF 14%, *SUZ12* c.1150_1151delAG VAF 20%	
44B						Post therapy			Non-informative			
44C						Refractory disease			Non-informative			
45A	Undifferentiated embryonal sarcoma of the liver	Initial diagnosis	Localized	Liver	F/6	At diagnosis-post surgery	YES	Abnormal	Non-informative	*TP53* c.844C>T, p.Arg282Trp VAF 9%	Not detected	
46A	Undifferentiated embryonal sarcoma of the liver	Initial diagnosis	Localized	Liver	M/14	At diagnosis	YES	Abnormal	Abnormal	*TP53* c.406C>T, p.Gln136* VAF 92%	*TP53* c.406C>T VAF 100%	
47A	Hepatoblastoma	Initial diagnosis	Localized		M/1	At diagnosis	YES	Non-informative	Non-informative	No clinically established variants		
48A	Hepatoblastoma	Initial diagnosis	Localized		M/2	At diagnosis	YES	Abnormal	Abnormal	No clinically established variants		
48B						At relapse			Abnormal			
49A	Hepatoblastoma	Initial diagnosis	Localized		M/15m	At diagnosis	YES	Abnormal	Abnormal	*CTNNB1* c.101G>A, p.Gly34Glu VAF 43%	Not detected	
49B						End of therapy			Non-informative			
50A	Wilms tumor	Initial diagnosis	Localized		F/6	At diagnosis-post surgery, during therapy	YES	Abnormal	Non-informative	*WT1* c.689_698del, p.His230Argfs*53 VAF 95%, *CTNNB1* c.133_135del, p.Ser45del VAF 84%	Not detected	
51A	Wilms tumor	Initial diagnosis	Localized		F/5	At diagnosis	YES	Abnormal	Non-informative	No clinically established variants		
52A	Clear cell sarcoma of Kidney	Initial diagnosis	Localized		M/18m	At diagnosis-post surgery, during therapy	YES	Non-informative	Non-informative	No clinically established variants		
53A	Wilms tumor	Initial diagnosis	Localized		M/2	At diagnosis	YES	Abnormal	Abnormal	No OncoKids report		
54A	Wilms tumor	Initial diagnosis	Localized		F/8	At diagnosis	YES	Abnormal	Abnormal	*SUFU* c.973_979delCCACCAA, p.Pro714Leufs*57 VAF 24%	Not detected	
55A	Wilms tumor	Initial diagnosis	Localized		M/3	At diagnosis-post surgery	YES	Abnormal	Abnormal	No clinically established variants		
56A	Wilms tumor	Initial diagnosis	Metastatic		M/3	At diagnosis	YES	Abnormal	Abnormal	*TP53* c.838A>G, p.Arg280Gly, TSPAN4-CD151 fusion VAF 58%	*TP53* c.838A>G VAF 14%	Not detected
56B						Post therapy			Non-informative			
56C						Pre Surgery			Non-informative			
57A	Wilms tumor	Relapse	Distant	Blateral lung metastases	F/11	At relapse	YES	Abnormal	Abnormal	*TP53* c.475_476delGC, p.Ala159Hisfs*21 VAF 89%	Not detected	
57B				Blateral lung metastases		At relapse (3rd relapse)			Abnormal			
58A	Wilms tumor	Relapse	Distant	Lung and Rib	F/9	At relapse	YES	Abnormal	Abnormal	No clinically established variants		
58B						Post surgery			Non-informative			
59A	Mesoblastic nephroma	Relapse	Distant	Bilateral lung metastases and liver metastases	F/2	At relapse	NO	NA	Abnormal	No OncoKids report		
60A	Wilms tumor	Relapse	Distant	Bilateral lung and liver	M/7	At relapse	YES	Abnormal	Abnormal	*TP53* c.743G>A, p.Arg248Gln VAF 76%, *FBXW7* c.1450A>G, p.Arg484Gly VAF 77%	*TP53* c.743G>A VAF 17%	
60B						Post therapy			Abnormal			
60C						Refractory disease			Abnormal			
61A	Clear Cell Sarcoma of Kidney	Relapse	Distant	Bilateral lung metastases	F/5	At relapse	NO	NA	Abnormal	No OncoKids report		
62A	Papillary thyroid carcinoma	Initial diagnosis	Metastatic		F/6	At diagnosis-post surgery	NO	NA	Non-informative	*TPR-NTRK1* fusion		Not detected
63A	Medullary thyroid carcinoma	Relapse	Distant	L neck and L supraclavicular nodes	F/10	At relapse	NO	NA	Non-informative	*RET* c.2753T>C, p.Met918Thr VAF 51%	Not detected	
64A	Germ cell tumor	Initial diagnosis	Localized		M/16	At diagnosis-post surgery	YES	Non-informative	Non-informative	No clinically established variants		
65A	Germ cell tumor	Initial diagnosis	Localized		M/16	At diagnosis-post surgery	NO	NA	Non-informative	No OncoKids report		

66A	Germ cell tumor	Initial diagnosis	Metastatic		M/14	At diagnosis	YES	Abnormal	Abnormal	No OncoKids report		
67A	Germ cell tumor	Relapse	Distant		M/16	At relapse-post surgery	NO	NA	Non-informative	No OncoKids report		
68A	Germ cell tumor	Initial diagnosis	Localized		F/9	At diagnosis	YES	Abnormal	Abnormal	*KIT* c.2447A>T, p.Asp816Val VAF 34%	*KIT* c.2447A>T VAF 20%	
68B						Post surgery			Non-informative			
68C						End of therapy			Non-informative			
69A	Germ cell tumor	Initial diagnosis	Localized		M/18	At diagnosis	YES	Abnormal	Abnormal	*TP53* c.581T>C, p.Leu194Pro VAF 58%, *KRAS* c.182A>G, p.Gln61Arg VAF 47%	*TP53* c.581T>C VAF 50%, *KRAS* c.182A>G VAF 75%	
69B						Post therapy			Abnormal			
69C						At progression			Abnormal			
70A	Germ cell tumor	Relapse	Local		M/16	At relapse-post surgery	NO	NA	Non-informative	No OncoKids report		
70B						Post surgery			Non-informative			
71A	Germ cell tumor	Initial diagnosis	Metastatic		M/18	At diagnosis-post surgery	YES	Abnormal	Abnormal	*KRAS* gene amplification		
71B						Post therapy			Non-informative			
71C						Post therapy			Non-informative			
71D						Pre surgery			Non-informative			
71E						Post surgery			Non-informative			
71F			Distant			Post therapy			Non-informative			
72A	Germ cell tumor	Relapse	Distant	Bilateral lung metastases and L kidney	M/18	At relapse	NO	NA	Abnormal	No OncoKids report		
73A	Germ cell tumor	Relapse	Distant	Bilateral lung metastases	M/18	At relapse	YES	Abnormal	Non-informative	No OncoKids report		
C1	Chiari malformation				F/15	NA	NA	NA	Non-informative	Not performed		
C2	Arachnoid cyst				M/11	NA	NA	NA	Non-informative	Not performed		
C3	Chiari malformation				F/7	NA	NA	NA	Non-informative	Not performed		
C4	Intraventricular hemorrhage				M/7w	NA	NA	NA	Non-informative	Not performed		
C5	Hydrocephalus				M/7	NA	NA	NA	Non-informative	Not performed		
C6	Chiari malformation				M/6	NA	NA	NA	Non-informative	Not performed		
C7	Chiari malformation				M/15	NA	NA	NA	Non-informative	Not performed		
C8	Neural tube defect				M/9	NA	YES (blood)	NA	chr10p loss	Not performed		
C9	Encephalocele, agenesis of the corpus callosum				F/9d	NA	YES (blood)	NA	chr1q loss	Not performed		
C10	Epilepsy				M/19	NA	NA	NA	Non-informative	Not performed		
C11	Hydrocephalus				M/2	NA	NA	NA	Non-informative	Not performed		
C12	Neuroglial malformation				M/12	NA	NA	NA	Non-informative	Not performed		
C13	Spinal syrinx				F/21	NA	NA	NA	Non-informative	Not performed		
C14	Epilepsy				M/18	NA	NA	NA	Non-informative	Not performed		
C15	Hydrocephalus				F/11	NA	NA	NA	Non-informative	Not performed		
C16	Hydrocephalus				F/15	NA	YES (blood)	NA	chr17q loss	Not performed		
C17	Hydrocephalus				F/14	NA	NA	NA	Non-informative	Not performed		
C18	Hydrocephalus				F/3m	NA	NA	NA	Non-informative	Not performed		
C19	Hydrocephalus VPS				M/9m	NA	NA	NA	Non-informative	Not performed		

NA: not available

VAF: variant allele frequency

Supplemental Table 1 Detailed patient characteristics Clinical data, characteristics, and results for 73 patients and 19 controls included in the study
